# Pulmonary Arterial Hypertension Specific Therapy in Patients with Combined Post- and Precapillary Pulmonary Hypertension

**DOI:** 10.1155/2018/7056360

**Published:** 2018-03-01

**Authors:** Hassan Alfraidi, Sultan Qanash, Zoheir Bshouty

**Affiliations:** ^1^Section of Pulmonary Medicine, Department of Medicine, University of Manitoba, Winnipeg, MB, Canada; ^2^Division of Cardiology, Department of Medicine, McGill University, Montreal, QC, Canada; ^3^King Saud Bin Abdulaziz University for Health Sciences, King Abdulaziz Medical City, Jeddah, Saudi Arabia

## Abstract

**Background:**

Specific therapy for patients with PAH is associated with good outcomes. Little is known about the effect of this treatment in patients with Cpc-PH (PAPm ≥ 25 mmHg, PAWP > 15 mmHg, DPG ≥ 7 mmHg, and/or PVR > 3 WU). This study evaluates the outcome of treating patients with Cpc-PH using PAH specific therapy.

**Methods:**

The primary outcome was survival. Secondary outcomes were WHO functional class and 6-minute walk distance (6-MWD).

**Results:**

Twenty-six patients with Cpc-PH (half with VHD and half with HF) received PAHST. Six patients did not tolerate treatment due to pulmonary edema. No predictors for treatment intolerance were identified. In twenty patients who tolerated the treatment, the mean WHO functional class improved from 2.70 ± 0.21 at initial assessment to 2.22 ± 0.21 (*p* < 0.04) and 2.06 ± 0.21 (*p* < 0.03) at 6 and 9 months, respectively. Mean 6-MWD improved from 276.0 ± 38.50 meters at initial assessment to 343.9 ± 22.99 meters (*p* < 0.04) and 364.6 ± 34.85 meters (*p* = 0.07) at 6 and 9 months, respectively. Twelve patients died during the follow-up period. Mean survival for all patients was 1279.7 ± 193.60 days.

**Conclusion:**

PAHST may be beneficial in the treatment of Cpc-PH (both short and long term). Prospective randomized controlled trials of PAHST in this population are needed to assess its potential efficacy.

## 1. Background

Pulmonary Hypertension (PH) is defined as Mean Pulmonary Artery Pressure (PAPm) ≥ 25 mmHg [1]. Pulmonary Arterial Hypertension (PAH) (also termed precapillary PH) is diagnosed when, in addition, PAWP is ≤15 mmHg [1]. Postcapillary PH is defined as PH due to left ventricular dysfunction (either myocardial and/or valvular) when PAWP is >15 mmHg [2, 3]. Isolated postcapillary PH (Ipc-PH) is said to be present when Diastolic Pressure Gradient (DPG = Diastolic PAP − PAWP) is <7 mmHg and PVR ≤ 3 WU [2].

Combined postcapillary and precapillary pulmonary hypertension (Cpc-PH) is a newly recognized entity in which PAWP > 15 mmHg and DPG ≥ 7 mmHg and/or PVR > 3 WU [2–4]. This group was previously referred to as having an “out-of-proportion” elevation in PAPm [3, 4] and represents almost 30% of the PAH patients. In spite of the above, it is worth noting that criteria for what constitutes “out of proportion” are not universally accepted [3, 4]. Some experts [5] have suggested a transpulmonary pressure gradient (TPG = PAPm − PAWP) of >12 mmHg in the setting of PAWP > 15 mmHg as a diagnostic criteria. However, using a fixed TPG or DPG [6, 7] without consideration of Cardiac Output and PAWP in a quite distensible vascular bed is physiologically unsound. In the normal pulmonary circulation, previous studies have shown that the rise in PAPm as a response to a rise in left atrial pressure (LAP) is nonlinear and is highly affected by mean alveolar pressure [8–10]. Raising LAP between zero and 10 mmHg causes hardly any rise in PAPm. As LAP further increases, the rise in PAPm (in proportion to the rise in LAP) increases gradually and approaches a 1 : 1 relationship only at very high LAP (in excess of 25 mmHg) [8–11]. Furthermore, the relationship between PAPm and LAP in the setting of PAH (and in various disease severities) is not known.

Data from the REVEAL registry has shown that PAH associated with PAWP > 19 mmHg has an increase mortality compared to PAWP < 15 mmHg [12, 13]. Despite the cardinal component of PAH (precapillary), those patients remain untreated with PAHST due to concerns extrapolated from outcomes in the management of patients with heart failure [14, 15]. PAHST is confined mainly to PAH, which mandates the absence of elevated left atrial pressure reflected by PAWP < 15 mmHg [1]. In this study, we evaluated the outcome of treating Cpc-PH with PAHST.

## 2. Methods

### 2.1. Study Design

This is a retrospective descriptive study of data obtained from the pulmonary hypertension registry (PHR) at the University of Manitoba (Health Science Centre, Winnipeg, MB). The data in the PHR is collected prospectively from a patient's initial assessment throughout every clinic visit following treatment initiation (approximately every 3 months). The PHR includes comprehensive data related to demographics, comorbidities, prior and concomitant medications, all required investigations to establish a proper diagnosis including invasive and noninvasive hemodynamic parameters, WHO functional class, and 6-minute walk distance (6-MWD). Data collection into the PHR was approved by the University of Manitoba research ethics board.

### 2.2. Assessments and Definitions

The definition of PH used in the study was based on the latest consensus [5th World Symposium of Pulmonary Hypertension, WSPH, [1]]. PH was defined as resting PAPm ≥ 25 mmHg [1]. All patients underwent right heart catheterization to confirm the diagnosis. All patients with Cpc-PH (as defined above), in addition to having TPG > 12 mmHg vascular compromise > 55% (see below), were included in the study.

We have previously developed a model of the normal pulmonary circulation, which was later adapted to simulate PAH [16]. A set of pulmonary hemodynamic data is entered into the model and through iterations, the model calculates the degree of vascular compromise (measured as percent of pulmonary arterial luminal loss at the precapillary level) that is associated with the entered data [17]. This model is applied to the hemodynamic data of all patients treated at our center. When applying model predictions to all patients in our database with PAH (PAPm ≥ 25 mmHg and PAWP ≤ 15 mmHg), vascular compromise was higher than 55%. This is why a vascular compromise threshold > 55% was used in addition to the criteria of Cpc-PH described above.

PH severity was assessed using WHO functional class and 6-MWD. WHO functional class and 6-MWD were assessed at baseline and at least every 3 months thereafter for one year. After one year, WHO functional class and 6-MWD were assessed every 3–6 months until being lost to follow-up or death. The 6-MWD was performed by trained and certified pulmonary function laboratory technicians according to the American Thoracic Society (ATS) guidelines [18]. Predicted distance for each individual was calculated using reference equations published by Enright and Sherrill in 1998 [19]. The primary end point was survival. Secondary end points included WHO functional class and 6-MWD 6 and 9 months following treatment initiation.

### 2.3. Treatment Protocol

All patients were initiated on monotherapy with either an endothelin receptor antagonist (ERA) or a phosphodiesterase-5 (PDE-5) inhibitor. Intolerance to a medication prompted a switch to another medication either within the same group or within another group dependent on the cause of the intolerance. The treatment protocol used in the PH clinic is a stepwise approach based on the goal-oriented treatment of PAH [20] as described by Hoeper et al. (and in conjunction with 5th WSPH). Patients who reported inadequate improvement in symptoms or did not achieve 380 meters within 3 to 6 months of monotherapy were stepped up to combination therapy. A drop in 6-MWD of greater than 50 meters was considered clinically significant when assessing individual patients. NYHA classification for dyspnea was recorded, although patients' lack of subjective improvement in symptoms was considered the more relevant clinical response.

### 2.4. Statistical Analysis

The statistical package used was Statistica. Analysis of variance for repeated measures (ANOVAR) with Tukey's test for specific comparisons was used to compare 6-MWD at 6 and 9 months after treatment initiation to baseline. Friedman ANOVA followed by Wilcoxon signed-rank tests with Bonferroni correction were used to compare WHO functional class at 6 and 9 months after treatment initiation to baseline. Unpaired *t*-test was used to compare parametric demographics (chi-square was used for nonparametric data), baseline hemodynamics, and functional capacity between patients who did and those who did not tolerate PAHST. Cox proportional hazards regression was used to identify predictors of survival.

## 3. Results

### 3.1. Study Population

Between July 2001 and December 2015, twenty-six patients with Cpc-PH underwent initial assessment. Six patients developed pulmonary edema within the first 3 months of treatment that was deemed due to PAHST and were excluded from the short- and long-term outcome analysis. Of the twenty patients who tolerated PAHST, half had a valvular heart disease (VHD) while the other half suffered from heart failure (HF). At our center, patients with VHD are considered for PAHST only if they have persistent PAH or Cpc-PH following valve repair and/or replacement with no evidence of significant residual stenosis and/or regurgitation (of either aortic or mitral valve). In the other ten patients who were diagnosed with HF, six had diastolic dysfunction (DD) with preserved CO and four had systolic dysfunction (SD). Of the six patients who did not tolerate PAHST, four had VHD and two had HF (one DD and one SD). Of the twenty patients who tolerated treatment, 13 (65%) were females and 7 (35%) were males. The mean age (±SE) for all patients was 64.7 ± 2.93 years (females 63.0 ± 2.65 and males 67.8 ± 7.01). Basic demographics, invasive hemodynamic data, baseline, 3-month, 6-month, and 9-month WHO, and 6-MWD are shown in Table 1. Data are shown as mean and standard error (SE).

### 3.2. Treatment

Out of the twenty patients who tolerated treatment, fifteen patients were treated with Bosentan monotherapy, two patients were on Sildenafil monotherapy, and three patients were treated with a combination of both. None of these patients required a change in treatment during the observation period. The six patients who did not tolerate treatment all received Bosentan. All discontinuations were due to pulmonary edema and none because of liver function abnormalities. All patients who tolerated treatment received treatment for at least 6 months and eighteen patients were treated for at least 9 months.

### 3.3. Right Heart Catheterization (RHC) Data

All patients underwent RHC. Mean (±SE) pulmonary artery pressure was 51.3 ± 2.71 mmHg, mean PAWP was 23.6 ± 0.84 mmHg, mean TPG was 27.7 ± 2.53 mmHg, mean DPG was 8.3 ± 1.65 mmHg, and mean PVR was 6.5 ± 0.78 WU. Based on model predictions, mean vascular compromise was 71.2 ± 1.20%. The rest of the hemodynamic data is shown in Table 1.

### 3.4. WHO Functional Class

**Figure 1:**
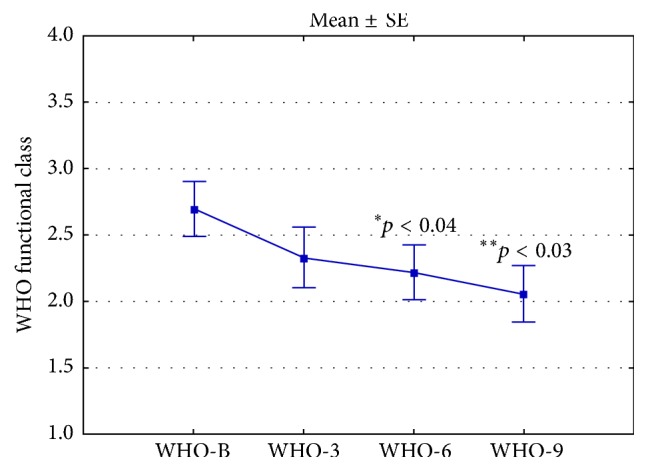
WHO functional class at baseline, 3 months, 6 months, and 9 months after initiation of PAHST.

Mean (±SE) WHO functional class for all patients at initial assessment was 2.70 ± 0.21. Mean WHO functional class at 6 and 9 months after treatment initiation was 2.22 ± 0.21 (*p* < 0.04, compared to baseline) and 2.06 ± 0.21 (*p* < 0.03), respectively (Figure 1).

### 3.5. 6-MWD

**Figure 2:**
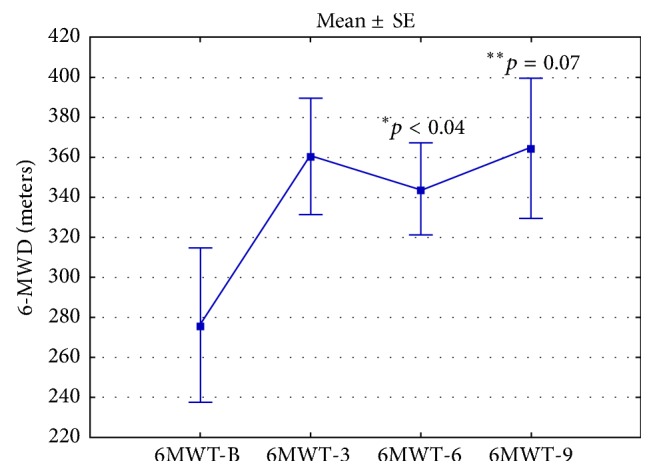
6-minute walk test distance (6-MWT) at baseline, 3 months, 6 months, and 9 months after initiation of PAHST.

Mean (±SE) 6-MWD for all patients at initial assessment was 276.0 ± 38.50 meters (56.4 ± 6.84% predicted). Mean 6-MWD distances at 6 and 9 months after treatment initiation were 343.9 ± 22.99 meters (72.8 ± 3.58% predicted) (*p* < 0.04, compared to baseline) and 364.6 ± 34.85 meters (74.1 ± 4.26% predicted) (*p* = 0.073, compared to baseline), respectively (Figure 2).

### 3.6. Survival

**Figure 3:**
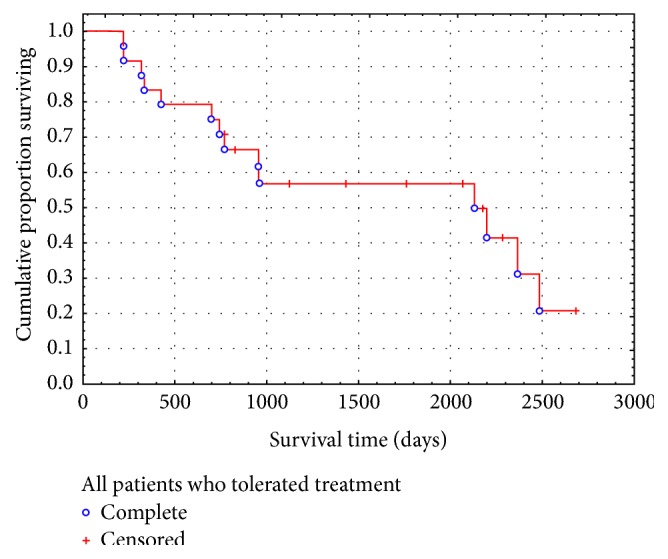
Survival rate of the study population receiving PAHST up to 6 years.

Out of twenty patients, 12 patients died during the follow-up period (6 years). Mean (±SE) survival for all patients was 1279.7 ± 193.6 days (1383.2 ± 250.4 days for females and 1087.4 ± 311.0 days for males). Kaplan Meier survival curve showed slightly lower survival for patients with Cpc-PH when compared to patients with pure PAH. A survival rate of 80%, 75%, and 53% at one, two, and three years (Figure 3) was noted, respectively. During the same period of observation, one, two, and three years survival rates in patients with pure PAH in our registry were 89%, 79%, and 67%, respectively. No predictors of survival could be identified among the parameters shown in Table 1.

## 4. Discussion

In the absence of mitral stenosis, PAWP and LVEDP are both considered accurate surrogates for left atrial pressure [21, 22]. Chronic elevations in left arterial pressure are associated with more than pressure related effects on the pulmonary circulation [23]. Vascular remodeling [24] and endothelial dysfunction [25], resembling PAH, have been described. Combined precapillary and postcapillary PH is a newly recognized entity which has specific hemodynamic parameters that include a PAWP > 15 mmHg, DPG ≥ 7 mmHg, and/or PVR > 3 WU [2–4, 7]. These patients tend to be obese, have more comorbidities, and perform less in 6-MWD [12, 13]. Data from the REVEAL registry has shown that patients with PAH associated with PAWP > 19 mmHg have a higher mortality rate over 2 years when compared to patients with PAWP < 15 mmHg [13].

In the present study, we combined the criteria of Cpc-PH, TPG > 12 mmHg, and we used model predictions of vascular compromise > 55% to ensure that the observed PAPm is out of proportion to the associated rise in PAWP. Calculated mean vascular compromise in both groups (those who tolerated and those who did not tolerate treatment) was higher (71.2 and 71.4%, resp.) than patients with pure PAH (55%) in our database suggesting that these patients had significant precapillary vasculopathy.

Several trials were performed to test the efficacy of PAHST on the treatment of heart failure [13–15]. In these studies, increased mortality and safety concerns have outweighed the beneficial effects on hemodynamics, exercise tolerance, and quality of life [14, 26, 27]. The effect of PAHST on Cpc-PH has been studied with variable outcomes [28–31]. The REVEAL registry has included patients with PAWP 15–18 mmHg. Compared to isolated PAH, PAH with PAWP > 19 mmHg is associated with increased mortality [13]. A recent trial in patients with PH and DPG > 20 mmHg who were treated with PAHST had promising results [32].

In the present study, twenty of twenty-six patients with Cpc-PH tolerated PAHST. In those patients, we noted some improvement in WHO functional class and 6-MWD at 6 and 9 months after treatment initiation (Figures 1 and 2).

Pulmonary Arterial Hypertension Specific Therapy has resulted in increased survival at one, two, and three years in patients with PAH (93%, 75% and 66%, resp.) [33]. It is comforting to see that our survival rates in patients with PAH (89%, 79% and 67%, resp.) are comparable to the published survival rates. In our observational study, patients with Cpc-PH who received and tolerated PAHST had slightly lower survival rates of 80%, 75%, and 53%, respectively (Figure 3).

All six patients who did not tolerate PAHST had their treatment discontinued because of developing pulmonary edema and none of these patients sustained end-organ damage or died due to this treatment trial. We could not identify, among the monitored factors, predictors that would identify patients who are more likely to tolerate PAHST.

This study has many limitations including its retrospective design, small sample size, and lack of a control group. The lack of a control group is a legitimate concern as we have no way of predicting whether not treating these patients would have resulted in similar or even better outcomes, although the sustained improvement in WHO functional class and 6-MWD led us to believe that we have not caused harm. It may be considered a pilot study or case series that is alerting us to a group of patients with Cpc-PH, some of whom may benefit from PAHST. Randomized controlled trials, on a much larger scale, are needed to answer the question of benefit and survival in this group of patients. These studies may also help identify predictors of good outcome that would lead to a better selection of patients who are likely to benefit from PAHST.

## 5. Conclusion

Pulmonary Arterial Hypertension Specific Therapy may be beneficial in the treatment of combined post- and precapillary pulmonary hypertension (both short and long term). Prospective randomized controlled trials of PAHST in this population are needed to assess its potential efficacy.

## Figures and Tables

**Table 1:** Descriptive statistics of patients enrolled in the study.

Variable	Patients who tolerated PAHST^*∗*^			Patients who did not tolerate PAHST^*∗*^		
	Number of patients	Mean	SE	Number of patients	Mean	SE
Age	20	64.6	2.93	6	67.4	4.61
CCI	20	3.80	0.36	6	4.0	0.37
PAPm	20	51.3	2.71	6	48.8	3.45
PAWP	20	23.6	0.84	6	27.0	2.80
TPG	20	27.7	2.53	6	21.8	2.27
PVR	19	6.5	0.78	6	7.14	2.47
DPG	20	8.3	1.65	6	4.2	2.06
CO	19	4.46	0.21	6	3.68	0.25
Vascular compromise	19	71.2	1.20	6	71.4	2.47

WHO-baseline	20	2.70	0.21	6	3.00	0.37
WHO-3 months	18	2.33	0.23			
WHO-6 months	18	2.22	0.21			
WHO-9 months	16	2.06	0.21			
6-MWD-baseline	23	276.0	38.50	6	268.0	50.52
6-MWD-3 months	17	360.5	29.01			
6-MWD-6 months	17	343.9	22.99			
6-MWD-9 months	15	364.6	34.85			
6-MWD (% pre)-baseline	23	56.4	6.83	6	58.3	8.99
6-MWD (% pre)-3 months	17	72.6	3.82			
6-MWD (% pre)-6 months	17	72.8	3.58			
6-MWD (% pre)-9 months	15	74.1	4.26			
Survival (in days)	20	1350.8	173.44			

^*∗*^PAHST = Pulmonary Arterial Hypertension Specific Therapy, CCI = Charlson Comorbidities Index, PAPm = Mean Pulmonary Artery Pressure (mmHg), PAWP = Pulmonary Arterial Wedge Pressure (mmHg), CO = Cardiac Output (L/min), TPG = Transpulmonary Gradient (mmHg), DPG = Diastolic Pressure Gradient (mmHg), PVR = Pulmonary Venous Resistance (WU), WHO = World Health Organization functional class, 6-MWD = 6-minute walk distance (meters), and SE = standard error.

## Data Access

The data is available in electronic form (Excel sheets) and in the PAH Database (PHMS) at the Division of Pulmonary Medicine, Department of Medicine, University of Manitoba.

## References

[1] Hoeper M. M., Bogaard H. J., Condliffe R. (2013). Definitions and diagnosis of pulmonary hypertension. *Journal of the American College of Cardiology*.

[2] Galiè N., Humbert M., Vachiery JL., Gibbs S., Lang I. (2015). 2015 ECS/ERS Guidelines for the diagnosis and treatment of pulmonary hypertension. *European Heart Journal*.

[3] Guazzi M., Borlaug B. A. (2012). Pulmonary hypertension due to left heart disease. *Circulation*.

[4] Vachiéry J.-L., Adir Y., Barberà J. A. (2013). Pulmonary hypertension due to left heart diseases. *Journal of the American College of Cardiology*.

[5] Naeije R., Vachiery J.-L., Yerly P., Vanderpool R. (2013). The transpulmonary pressure gradient for the diagnosis of pulmonary vascular disease. *European Respiratory Journal*.

[6] Gerges C., Gerges M., Lang M. B. (2013). Diastolic pulmonary vascular pressure gradient: A predictor of prognosis in "out-of-proportion" pulmonary hypertension. *CHEST*.

[7] Tampakakis E., Leary P. J., Selby V. N. (2015). The diastolic pulmonary gradient does not predict survival in patients with pulmonary hypertension due to left heart disease. *JACC: Heart Failure*.

[8] Chaliki H. P., Hurrell D. G., Nishimura R. A., Reinke R. A., Appleton C. P. (2002). Pulmonary venous pressure: Relationship to pulmonary artery, pulmonary wedge, and left atrial pressure in normal, lightly sedated dogs. *Catheterization and Cardiovascular Interventions*.

[9] Albert R. K., Lamm W. J. E. (2003). Left atrial pressure can be accurately transmitted to the pulmonary artery despite Zone 1 conditions. *American Journal of Respiratory and Critical Care Medicine*.

[10] Saouti N., Westerhof N., Postmus P. E., Vonk-Noordegraaf A. (2010). The arterial load in pulmonary hypertension. *European Respiratory Review*.

[11] Ilsar R., Bailey B. P., Dobbins T. A., Celermajer D. S. (2010). The Relationship Between Pulmonary Artery and Pulmonary Capillary Wedge Pressure for the Diagnosis of Pulmonary Vascular Disease. *Heart, Lung and Circulation*.

[12] Badesch D. B., Raskob G. E., Elliott C. G. (2010). Pulmonary arterial hypertension: baseline characteristics from the REVEAL registry. *CHEST*.

[13] Frost A. E., Farber H. W., Barst R. J., Miller D. P., Elliott C. G., McGoon M. D. (2013). Demographics and outcomes of patients diagnosed with pulmonary hypertension with pulmonary capillary wedge pressures 16 to 18 mm Hg: Insights from the REVEAL registry. *CHEST*.

[14] Califf R. M., Adams K. F., McKenna W. J., et al (1997). A randomized controlled trial of epoprostenol therapy for severe congestive heart failure: the Flolan International Randomized Survival Trial (FIRST). *American Heart Journal *.

[15] Packer M., McMurray J., Massie B. M. (2005). Clinical effects of endothelin receptor antagonism with bosentan in patients with severe chronic heart failure: Results of a pilot study. *Journal of Cardiac Failure*.

[16] Bshouty Z., Younes M. (1990). Distensibility and pressure-flow relationship of the pulmonary circulation. II. Multibranched model. *Journal of Applied Physiology*.

[17] Bshouty Z. (2012). Vascular compromise and hemodynamics in pulmonary arterial hypertension: Model predictions. *Canadian Respiratory Journal*.

[18] (2002). ATS statement: guidelines for the six-minute walk test. *American Journal of Respiratory and Critical Care Medicine*.

[19] Enright P. L., Sherrill D. L. (1998). Reference equations for the six-minute walk in healthy adults. *American Journal of Respiratory and Critical Care Medicine*.

[20] Hoeper M. M., Markevych I., Spiekerkoetter E., Welte T., Niedermeyer J. (2005). Goal-oriented treatment and combination therapy for pulmonary arterial hypertension. *European Respiratory Journal*.

[21] Flores E. D., Lange R. A., Hillis L. D. (1990). Relation of mean pulmonary arterial wedge pressure and left ventricular end-diastolic pressure. *American Journal of Cardiology*.

[22] De Oliveira R. K. F., Ferreira E. V. M., Ramos R. P. (2014). Usefulness of pulmonary capillary wedge pressure as a correlate of left ventricular filling pressures in pulmonary arterial hypertension. *The Journal of Heart and Lung Transplantation*.

[23] Dayeh N. R., Ledoux J., Dupuis J. (2016). Lung Capillary Stress Failure and Arteriolar Remodelling in Pulmonary Hypertension Associated with Left Heart Disease (Group 2 PH). *Progress in Cardiovascular Diseases*.

[24] West J. B., Mathieu-Costello O. (1995). Vulnerability of pulmonary capillaries in heart disease. *Circulation*.

[25] Farrero M., Blanco I., Batlle M. (2014). Pulmonary hypertension is related to peripheral endothelial dysfunction in heart failure with preserved ejection fraction. *Circulation: Heart Failure*.

[26] Lewis G. D., Shah R., Shahzad K. (2007). Sildenafil improves exercise capacity and quality of life in patients with systolic heart failure and secondary pulmonary hypertension. *Circulation*.

[27] Botha P., Parry G., Dark J. H., MacGowan G. A. (2009). Acute Hemodynamic Effects of Intravenous Sildenafil Citrate in Congestive Heart Failure: Comparison of Phosphodiesterase Type-3 and -5 Inhibition. *The Journal of Heart and Lung Transplantation*.

[28] Bonderman D., Ghio S., Felix S. B. (2013). Riociguat for patients with pulmonary hypertension caused by systolic left ventricular dysfunction: A phase IIb double-blind, randomized, placebo-controlled, dose-ranging hemodynamic study. *Circulation*.

[29] Jiang R., Wang L., Zhu C.-T. (2015). Comparative effectiveness of sildenafil for pulmonary hypertension due to left heart disease with HFrEF. *Hypertension Research*.

[30] Hoendermis E. S., Liu L. C. Y., Hummel Y. M. (2015). Effects of sildenafil on invasive haemodynamics and exercise capacity in heart failure patients with preserved ejection fraction and pulmonary hypertension: A randomized controlled trial. *European Heart Journal*.

[31] Kaluski E., Cotter G., Leitman M. (2008). Clinical and hemodynamic effects of bosentan dose optimization in symptomatic heart failure patients with severe systolic dysfunction, associated with secondary pulmonary hypertension - A multi-center randomized study. *Cardiology*.

[32] Gerges C., Gerges M., Skoro-Sajer N. (2016). Hemodynamic thresholds for precapillary pulmonary hypertension. *CHEST*.

[33] Thenappan T., Shah S. J., Rich S., Tian L., Archer S. L., Gomberg-Maitland M. (2010). Survival in pulmonary arterial hypertension: A reappraisal of the NIH risk stratification equation. *European Respiratory Journal*.

